# Advances in gene editing tools for four typical Gram-positive bacteria

**DOI:** 10.3389/fmicb.2026.1882312

**Published:** 2026-06-26

**Authors:** Zishan Liang, Zhengyu Li, Chunming Li, Yongqing Zhao, Junlin Liu, Jiao Zhang

**Affiliations:** 1 Biology Engineering College, Northwest Minzu University, Lanzhou, China; 2 Key Laboratory of Biotechnology and Bioengineering of State Ethnic Affairs Commission, Biomedical Research Center, Northwest Minzu University, Lanzhou, China; 3 Engineering Research Center of Key Technology and Industrialization of Cell-based Vaccine, Ministry of Education, Biomedical Research Center, Northwest Minzu University, Lanzhou, China; 4 Gansu Tech Innovation Center of Animal Cell, Biomedical Research Center, Northwest Minzu University, Lanzhou, China

**Keywords:** CRISPR-Cas, *Lactobacillus plantarum*, *Lactococcus lactis*, *Bacillus subtilis*, *Corynebium glutamicum*

## Abstract

Gram-positive bacteria serve as important chassis microorganisms in synthetic biology, industrial fermentation, and probiotic development. The rapid advancement of gene editing technologies has provided critical technical support for the iterative construction and functional validation of engineered strains. However, due to factors such as cell wall structure, differences in genetic backgrounds, and tool compatibility, the development and editing efficiency of gene editing systems for Gram-positive bacteria still face many challenges. This review focuses on four representative Gram-positive bacterial species-*Lactobacillus plantarum*, *Lactococcus lactis*, *Bacillus subtilis*, and *Corynebacterium glutamicum*-and traces the evolution and current state of their editing tools, from traditional homologous recombination to CRISPR-Cas9, base editors, and large-fragment integration tools. On this basis, we summarize the common challenges and corresponding strategies concerning host repair capacity, tool compatibility, and inherent limitations of editors in these four bacterial species, and propose recommendations for tool selection based on different application scenarios. This review aims to provide a technical reference for gene editing studies of the above-mentioned bacterial species. Although the conclusions cannot be directly extended to all Gram-positive bacteria, the common issues summarized here may inform the development of gene editing tools for other Gram-positive bacteria.

## 1 Introduction

Gram-positive bacteria are important chassis organisms in synthetic biology and microbial metabolic engineering, playing an irreplaceable role in fields such as food fermentation, bulk chemical synthesis, production of high-value pharmaceutically active substances, and live biotherapeutic products. Among these, representative bacterial species such as *Lactobacillus plantarum*, *Lactococcus lactis*, *Bacillus subtilis*, and *Corynebacterium glutamicum* have become key model bacterial species in industrial biotechnology and basic research due to their clear genetic backgrounds, high biosafety, and well-established fermentation processes (Liu and Yu, 2025; Zhan et al., 2024; Zhang et al., 2026). Efficient and precise gene editing technologies are core tools for achieving targeted genome modification and metabolic pathway optimization in these bacterial species. Therefore, developing editing tools adapted to different Gram-positive bacteria is of great significance for promoting their large-scale industrial applications.

Early genome modification of Gram-positive bacteria mainly relied on homologous recombination (HR) (Biswas et al., 1993) and site-specific recombination systems such as Cre/*loxP* (Xin et al., 2018). Although these methods enable gene knockout and insertion, they generally suffer from cumbersome procedures, long operation cycles, low editing efficiency, and high false-positive rates, which limit their ability to meet the demands for rapid iterative editing and precise modification (Leenay et al., 2019; Westbrook et al., 2016). The discovery and rapid iteration of the CRISPR-Cas system have advanced gene editing technologies into a new stage. CRISPR nuclease tools such as Cas9, Cpf1, and MAD7 have been gradually adapted to various Gram-positive bacteria, effectively broadening the scope of gene editing applications and improving editing efficiency (Altenbuchner, 2016; Jiang et al., 2017). Relying on technologies such as base editors (BEs), recombinase engineering, and CRISPR-associated transposase systems (CAST), efficient single-base substitution, simultaneous multiplex editing, and site-specific integration of large DNA fragments have now been achieved (Liu et al., 2019; Yang et al., 2023; Yu et al., 2020).

Despite significant progress in the above areas, the overall development of gene editing systems for Gram-positive bacteria still lags behind that for Gram-negative model bacteria such as *Escherichia coli*, which has a very well-defined genetic background. The development of cutting-edge editing technologies is typically first validated and iterated in *E. coli* (Chao and Hu, 2022; Roberts et al., 2025) before being gradually adapted to various Gram-positive bacteria. However, Gram-positive bacteria exhibit substantial variability in transformation efficiency, CRISPR system compatibility (e.g., host toxicity of Cas effector proteins), and host recombination and repair capabilities, making it difficult to transfer many new technologies. Moreover, common problems such as limited multiplex editing, low efficiency of large-fragment integration, and off-target effects continue to constrain the further development of gene editing technologies in Gram-positive bacteria (Leenay et al., 2019; Jiang et al., 2017; Liu et al., 2019; Mitsunobu et al., 2025).

**Figure 1:**
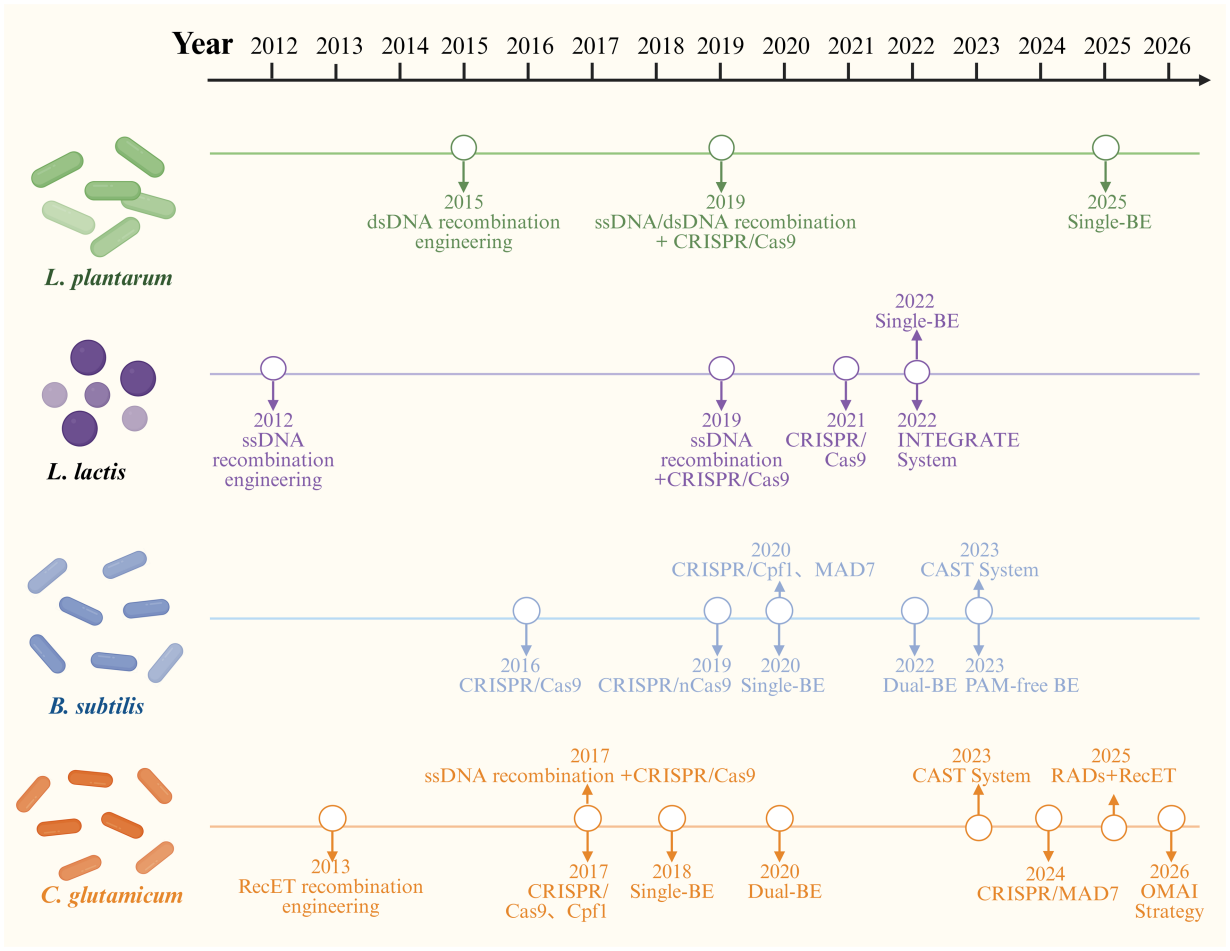
Development history of gene editing tools in four representative Gram-positive bacteria. The horizontal axis represents the years 2012–2026, and different colors represent different bacterial species. The figure marks the time points at which each gene-editing tool was first developed.

**Figure 2:**
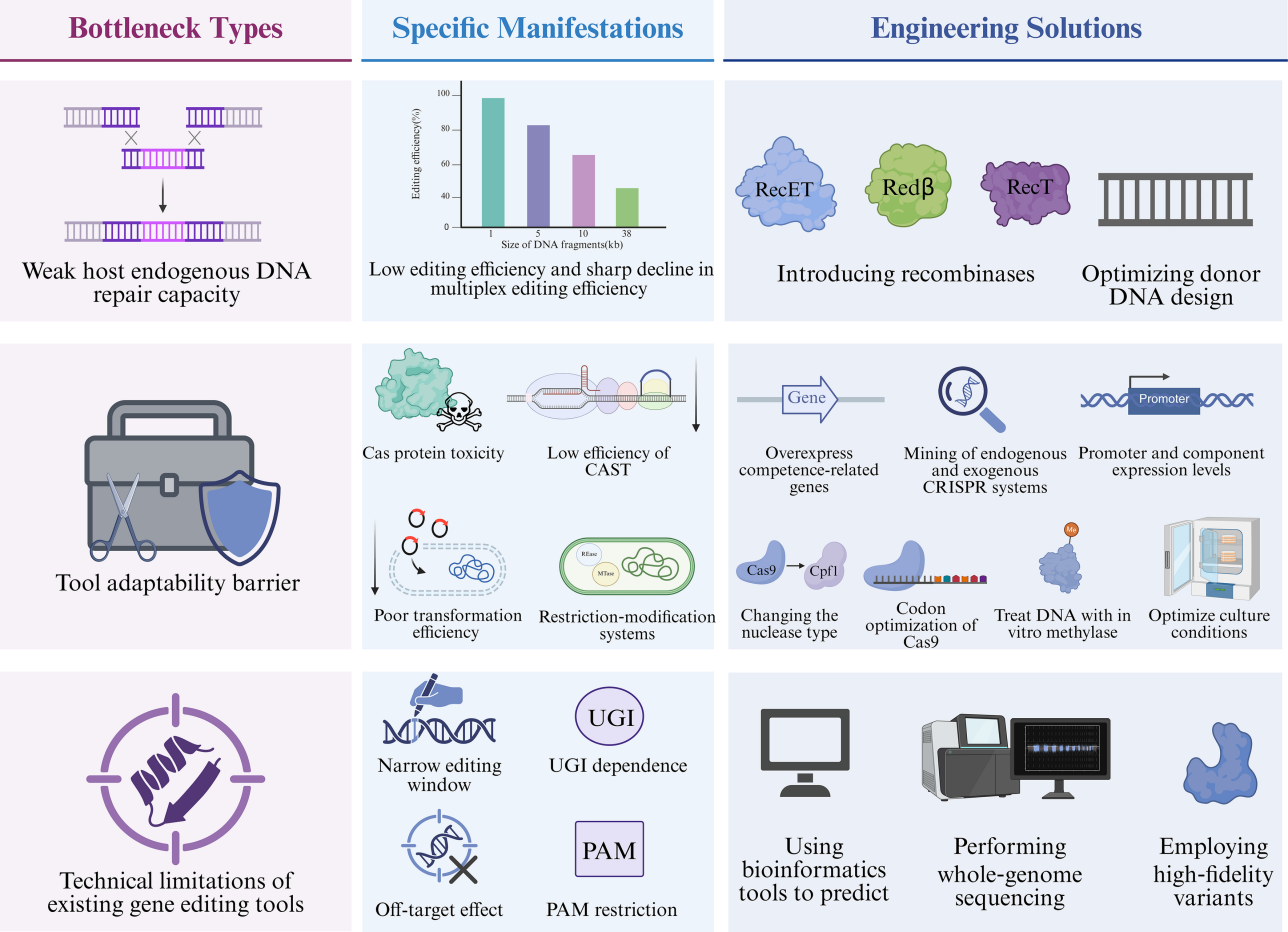
Common technical bottlenecks and corresponding engineering strategies for gene editing. The three columns indicate, from left to right: (1) three shared bottleneck types in four representative Gram-positive bacteria (weak endogenous DNA repair, tool adaptability barriers, and technical limitations of existing tools); (2) specific manifestations (e.g., low editing efficiency, Cas protein toxicity, low efficiency of CAST, low transformation efficiency, off-target effect, PAM restriction); and (3) corresponding engineering solutions (e.g., introducing recombinases, optimizing donor DNA design, mining CRISPR systems, using bioinformatics tools to predict).

**Figure 3:**
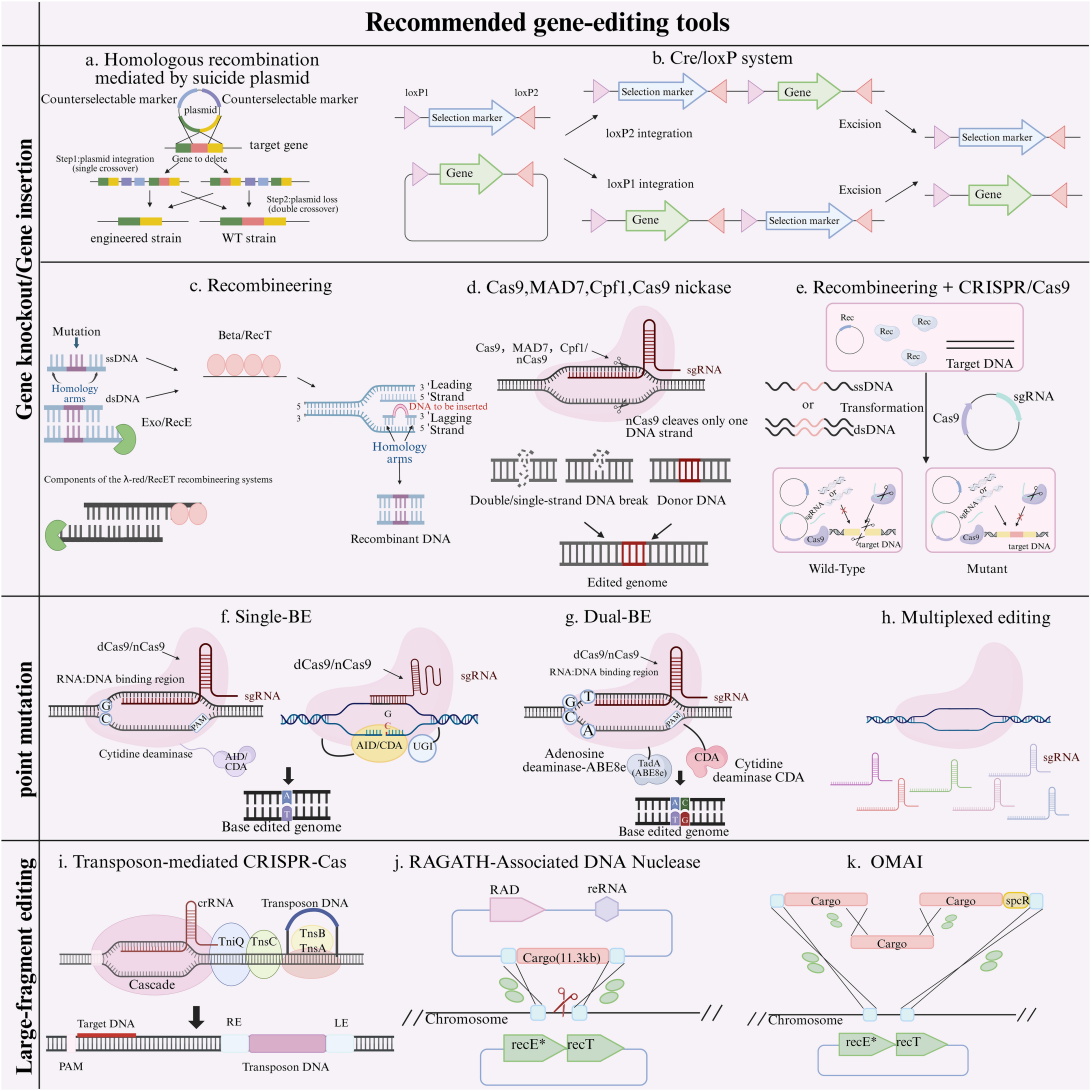
Tool selection strategies for different editing scenarios. All editing strategies are divided into three functional modules: gene knockout/insertion, point mutation, and large-fragment modification. **(a)** Suicide plasmid-based homologous recombination: Target gene manipulation relies on single/double crossover between counterselectable suicide plasmids and wild-type genomes. **(b)** Cre/loxP site-specific recombination: loxP-flanked selection markers are integrated into the chromosome and subsequently excised by Cre recombinase for marker-free editing. **(c)** λ-Red/RecET recombineering: Phage Exo/RecE and Beta/RecT recombinases utilize short-homology-arm ssDNA/dsDNA donors to drive genome modification. **(d)** CRISPR (Cas9, MAD7, Cpf1) and nCas9 nickase: sgRNA-guided nucleases introduce DSBs or SSBs, and subsequent HDR with donor DNA yields edited strains. **(e)** Recombineering combined with CRISPR/Cas9: RecET/λ-Red mediates donor DNA recombination, while Cas9 eliminates unmodified wild-type cells to enrich mutants. **(f)** Single base editor (Single-BE): dCas9/nCas9 fused with cytidine deaminase (CBE) mediates C → T conversion, and fusion with adenosine deaminase (ABE) achieves A → G substitution; fused UGI inhibits base excision repair to retain edited bases. **(g)** Dual base editor (Dual-BE): dCas9/nCas9 fused with both adenosine and cytidine deaminases achieves concurrent A-to-G and C-to-T single-base substitutions. **(h)** Multiplex editing: Multiple sgRNAs are delivered to target different loci concurrently, realizing parallel modification at multiple sites. **(i)** CRISPR-associated transposon editing: CRISPR-Cascade guides transposon complexes to integrate large cargo DNA at PAM-proximal target sites without homology arms. **(j)** RAGATH system: recE*/recT recombinases cooperate with RAGATH nucleases to integrate large exogenous DNA cargos into chromosomes. **(k)** OMAI system: RecET variants assemble overlapping DNA fragments *in vivo* for one-step chromosomal integration, supporting ultra-large fragment insertion via iterative editing.

Most existing reviews focus on a single class or a single species [e.g., lactic acid bacteria (LAB) (Zhang et al., 2026; Shaposhnikov et al., 2025), *B. subtilis* (Cao et al., 2025; Wu et al., 2025; Zocca et al., 2022), *C. glutamicum* (Kim et al., 2023; Wang et al., 2021; Dudeja et al., 2025; Wang et al., 2023)], while few summarize the editing technologies across multiple typical Gram-positive bacteria and the common technical bottlenecks along with their solutions. To address this gap, this review focuses on *L. plantarum* and *L. lactis* (as representatives of LAB), *B. subtilis*, and *C. glutamicum*, outlining the development history (Figure 1), current application status, common bottlenecks, and solution strategies (Figure 2) of various editing tools in these different hosts. The aim is to provide researchers with a reference for selecting appropriate editing tools for the above-mentioned bacterial species (Figure 3) and to offer insights for the development of gene editing tools and synthetic biology applications in other Gram-positive bacteria.

## 2 Gene editing in LAB

*L. plantarum* and *L. lactis* are typical representatives of LAB. Due to their widespread presence in fermented foods and their Generally Recognized as Safe (GRAS) status, they have become important research subjects in food science, medicine, and synthetic biology (Chung et al., 2026). *L. plantarum*, with its good environmental adaptability and host interaction ability, shows great potential in probiotic development, novel vaccine delivery, and health food manufacturing (Zhang et al., 2025). *L. lactis*, as a classic model bacterial species for LAB genetic engineering, relies on flexible and controllable cloning and expression systems, and its application scenarios have expanded from traditional fermentation to high-value microbial cell factories (Song et al., 2017). Given the central roles of these two bacterial species, the following sections focus on them to introduce the gene editing tools and progress in LAB.

### 2.1 Traditional methods: HR and recombineering

In the early stages, gene editing in LAB mainly relied on RecA-dependent HR mediated by integrative plasmids and suicide vectors, achieving gene knockout and integration through double crossover, and counter-selection markers such as *upp* were used to increase the positive rate. However, these methods suffered from long operation cycles, low editing efficiency, high false-positive rates, and frequent retention of selection markers, making it difficult to meet the demands for scarless and high-throughput editing (Biswas et al., 1993; Douglas and Klaenhammer, 2011). To address these issues, recombineering based on phage recombinases was introduced into LAB. Among these, single-stranded DNA recombineering (ssDNA recombineering) relies on single-strand annealing proteins such as RecT or Redβ, which mediate recombination between oligonucleotides of approximately 90 bp and homologous regions on the chromosome, thereby enabling precise point mutations or short-deletion knockouts. In 2012, van Pijkeren et al. first established an ssDNA recombineering system in *L. lactis*, achieving targeted point mutations in different regions of genes such as *rpoB* and *ddl*, with editing efficiencies ranging from 0.4 to 19% (van Pijkeren and Britton, 2012). Subsequently, double-stranded DNA recombineering (dsDNA recombineering) was developed for the manipulation of larger DNA fragments. Yang et al. identified and utilized a recombination system mediated by *Lp_0640-41-42* (whose functions are similar to Gam, Beta, and Exo, respectively) derived from a prophage P1 element in *L. plantarum*. This system efficiently achieved gene knockout, large-fragment deletion, and exogenous gene knock-in using homology arms of approximately 1 kb, with significantly higher efficiency than the traditional RecA-dependent double-crossover method (Yang et al., 2015). However, this operon exhibits host specificity, for example, the recombination efficiency in strain *L. plantarum* WCFS1 is much higher than that in strain JDM1, possibly because the former has higher electroporation efficiency and the operon is silent in the latter (Yang et al., 2015; Xin et al., 2017). Therefore, more homologous genes in LAB need to be explored to broaden the technical applicability, and further optimization of experimental procedures is required.

### 2.2 Combination of CRISPR-Cas9 with recombineering

The advent of the CRISPR-Cas system has propelled LAB gene editing into a new era. CRISPR/Cas systems have been successfully applied in various LAB. Song et al. established a novel gene editing tool based on the CRISPR/Cas9 system in *L. lactis* NZ9000, enabling gene editing within 7 days, with knockout efficiencies of 8 to 50% for the *ldh* gene (Song et al., 2021). However, the DNA double-strand breaks (DSBs) generated by Cas9 are highly toxic to some LAB, certain *Lactobacillus* species are highly sensitive to DSBs, leading directly to cell death regardless of the presence of a repair donor. Even within the species *L. plantarum*, significant differences exist among strains in their endogenous recombination capacity required for DSB repair. Therefore, coupling CRISPR-Cas9 with recombinases can improve repair efficiency and thus enhance gene editing efficiency (Huang et al., 2019; Oh and van Pijkeren, 2014; Zhou et al., 2019; Zhou et al., 2023). Guo et al. combined ssDNA recombineering with CRISPR/Cas9 counter-selection to achieve efficient scarless gene deletion (50/100 bp) and insertion (34 bp) within 72 h in *L. lactis* NZ9000, with mutation efficiencies exceeding 75% for the *upp* and *galk* targets (Guo et al., 2019). Zhou et al. employed CRISPR/Cas9-assisted dsDNA recombineering to engineer *L. plantarum* WCFS1, efficiently achieving gene knockout, gene insertion, and point mutation (Zhou et al., 2019). Huang et al. coupled phage-derived RecE/T with CRISPR-Cas9 to construct a multi-functional toolbox comprising a recombination helper plasmid and a broad-host-range CRISPR-Cas9 editing plasmid, which enabled efficient editing of *L. plantarum* WCFS1 and *L. brevis* ATCC367. The RecE/T-assisted CRISPR-Cas9 toolbox achieved single-gene deletion within 7 days with efficiencies of 50–100%. Furthermore, chromosomal gene replacement of *Lp_0537* using the P23-pyruvate decarboxylase expression cassette was achieved with 35.7% efficiency (Huang et al., 2019). Zhou et al. developed a CRISPR/Cas9-based recombination system incorporating Rec/ET, using the *L. lactis*-derived plasmid pMG36e, and successfully applied it in *L. lactis*, significantly increasing the gene knockout efficiency to 91% (Zhou et al., 2023). Wiull et al. constructed an inducible RecE/T-assisted CRISPR-Cas9 dual-plasmid system in *L. plantarum* WCFS1 for site-specific gene integration (Wiull et al., 2025).

### 2.3 Base editing and CRISPR-associated transposases

Cas9 nickase (nCas9), obtained by inactivating the HNH domain (H840A mutation) or the RuvC domain (D10A mutation), generates a nick on only one strand under the guidance of sgRNA without creating a DSB, thereby offering higher safety (Davis and Maizels, 2014). BEs are fusions of nCas9 (or catalytically dead Cas9, dCas9) with a DNA deaminase, enabling precise point mutations without requiring DSBs or donor DNA templates (Anzalone et al., 2020; Arroyo-Olarte et al., 2021; Komor et al., 2016). Tian et al. constructed the first DSB-free multiplex editing system in *L. lactis*—the CRISPR deaminase-assisted BE (CRISPR-DBE), which includes a cytidine BE (CRISPR-cDBE) and an adenine BE (CRISPR-aDBE). CRISPR-cDBE efficiently achieved C → T mutations under sgRNA guidance and was successfully used to simultaneously inactivate multiple genes in *L. lactis* NZ9000 (Tian et al., 2022). Mitsunobu et al. established a Target-AID (activation-induced cytidine deaminase)-based CBE (cytidine base editor) system in *L. plantarum* WCFS1, using a LAB-*E. coli* shuttle plasmid backbone carrying optimized nCas9/dCas9, *Petromyzon marinus* cytidine deaminase (PmCDA1), uracil glycosylase inhibitor (UGI), and crRNA expression elements, and added a terminator at the crRNA end to significantly improve efficiency. In *L. plantarum*, the editing window of this system is located at positions 17–20 upstream of the PAM, and the single-target efficiency of C → T reached nearly 100%. Moreover, high-efficiency multiplex editing at three targets was achieved in a single round of transformation. In addition, the introduction of the NG-Cas9 variant expanded the targeting range to NG-type PAMs (Mitsunobu et al., 2025).

Large-fragment integration into the *L. lactis* genome is limited by low efficiency and the time-consuming property of HR. Although integration of exogenous fragments up to 5 kb has been achieved, the success rate is highly dependent on transformation efficiency (Casey et al., 1992; Van Zyl et al., 2019). Moreover, RecA-mediated HR efficiency is very low, even with counter-selection markers, construction of mutant strains can take up to 3 weeks and involves cumbersome procedures (Guo et al., 2019). Pechenov et al. adapted the INTEGRATE system (Rubin et al., 2022) to *L. lactis* IL1403 through vector design, promoter selection, experimental condition optimization, and sgRNA engineering, achieving editing efficiencies of 2 × 10^−4^ for 1 kb and 4 × 10^−5^ for 10 kb, while maintaining low off-target activity (Pechenov et al., 2022). However, this system has not yet been adapted to *L. plantarum*.

### 2.4 Summary

LAB are closely related to human life and play important roles in agriculture, industry, and medicine. This summary has outlined the technological evolution from HR, recombineering, and CRISPR-Cas9 coupling to base editing and CAST systems. Despite significant progress, several challenges remain: low efficiency of recombineering in certain strains, cytotoxicity of CRISPR-Cas9 in DSB-sensitive strains, limited editing windows and targeting ranges of BEs, and low efficiency of large-fragment integration and limited host adaptability of CAST systems.

At the application level, gene editing technologies have been widely used to enhance phage resistance, optimize flavor metabolic networks, increase the production of bioactive compounds such as exopolysaccharides and GABA, and improve probiotic traits including bile salt tolerance, acid tolerance, and intestinal colonization ability. Recombinant LAB can also serve as oral vaccine or therapeutic protein delivery vehicles for preventing viral enteritis and alleviating inflammatory bowel disease (Zhang et al., 2026; Dudeja et al., 2025; Mu et al., 2022; Roberts and Barrangou, 2020; Vaid et al., 2022). In the future, with the continuous iteration of CRISPR-Cas tools, LAB are expected to evolve from traditional fermentation starters to precisely designed live biotherapeutic products and sustainable bio-manufacturing platforms, bringing new opportunities for food science and human health.

## 3 Gene editing in *B. subtilis*

*B. subtilis* is a typical Gram-positive model bacterium and a GRAS microorganism. This bacterial species has a clear genetic background, is non-pathogenic, exhibits outstanding protein secretion capacity, and possesses a well-established fermentation technology system, making it an ideal host for the production of heterologous proteins such as small molecule compounds, bulk chemicals, enzymes, and functional peptides. *B. subtilis* has been widely used as a cell factory for producing chemicals, enzyme preparations, and antimicrobial materials in industry, agriculture, and medicine (Liu and Yu, 2025). Despite its broad industrial application prospects, the development of genetic tools for this bacterial species still lags behind that of mainstream production bacterial species such as *E. coli* and *Saccharomyces cerevisiae*.

### 3.1 Traditional methods

Early gene editing in *B. subtilis* mainly relied on HR-based methods, such as the use of positive selection markers (antibiotic resistance markers), counter-selection markers (*mazF*), or the Cre/*loxP* site-specific recombination system (Yan et al., 2008; Zhang et al., 2006). Although these methods enable single-gene knockout and scarless modification, they generally suffer from low editing efficiency, the need for multiple rounds of selection, and long operation cycles (Hong et al., 2018).

### 3.2 CRISPR-Cas systems for single-locus editing

The introduction of the CRISPR-Cas system marked a significant shift in gene editing technology for *B. subtilis*. Altenbuchner et al. constructed the single-plasmid system pJOE8999 and achieved knockout of a 25.1 kb large fragment in *B. subtilis* 168 with 89% efficiency, the knockout efficiency for the 4.1 kb pulcherrimin biosynthetic gene cluster was even higher, reaching 97% (Altenbuchner, 2016). Westbrook et al. developed a chromosome-integrated CRISPR/Cas9 toolkit in *B. subtilis*, achieving single- and double-gene mutation efficiencies of 100 and 85%, respectively, and successfully inserted the 2.9 kb hyaluronic acid biosynthetic operon into the chromosome with 69% efficiency (Westbrook et al., 2016). However, this method required the use of counter-selection markers to improve editing efficiency, and its utility for large-fragment gene knockout had not been validated. So et al. used a dual-plasmid system expressing SpCas9, dual sgRNAs, and a donor DNA template. By extending the cultivation time after plasmid transformation, the knockout efficiency for the 38 kb *pps* gene cluster reached 80%. Furthermore, in this system, the single-gene knockout efficiency for *spo0A* was nearly 100%, the point mutation efficiency for *sigE* was approximately 68%, and the site-specific insertion efficiency for the green fluorescent protein gene was approximately 97%, indicating that extended cultivation time effectively improved editing efficiency (So et al., 2017). Hao et al. integrated Cpf1 into the genome of *B. subtilis* 168 and used a single plasmid to express the sgRNA targeting *ppsC* and the repair homology arm; the established CCB-CIGE system achieved 80% knockout efficiency for the 38 kb *pps* gene cluster (Hao et al., 2020). However, the above methods required repeated elimination and transformation of plasmids for iterative editing, which was time-consuming. Zou et al. used a single-plasmid system expressing Cas9, an sgRNA targeting the gene of interest, a donor DNA, and an sgRNA targeting the replicon of the plasmid itself. After one round of editing, induction of the sgRNA targeting the replicon eliminated the plasmid, allowing a single editing cycle to be completed in as little as 2.5 days, thereby accelerating the genome editing cycle (Zou et al., 2022). MAD7 is a Cpf1-like nuclease and currently the smallest CRISPR-associated endonuclease used in *B. subtilis* (approximately 1,263 amino acids). Price et al. applied MAD7 to *B. subtilis*, achieving genome mutations using homology arms of approximately 1 kb, and found that the editing efficiencies of Cas9 and MAD7 were comparable. However, transformation efficiencies for both nuclease-expressing plasmids were low, yielding only 3–114 transformants (Price et al., 2020). Laforge et al. improved transformation efficiency by constructing a strain overexpressing competence gene. Integrating MAD7-gRNA into the genome and using temperature-controlled reversible inactivation of MAD7 enables efficient *in vivo* construction of mutant libraries targeting specific sequences (Laforge et al., 2025).

### 3.3 Multiplex gene editing based on CRISPR-Cas systems

As metabolic engineering shifts from single-gene modification to multi-gene coordinated regulation, the demand for simultaneous editing of multiple targets is increasingly prominent. Although *B. subtilis* naturally possesses a relatively high HR capacity (Bodmer and Ganesan, 1964), the simultaneous repair of multiple DSBs remains inefficient. To overcome this bottleneck, Liu et al. developed a nCas9-mediated gene editing system in *B. subtilis* 168. Compared with Cas9, nCas9 generates only single-strand breaks (SSBs), which are less damaging to the host. This system achieved knockout efficiencies of no less than 80% for fragments of 1–8 kb, insertion efficiencies of no less than 90% for fragments of 1–2 kb, and nearly 100% efficiency for site-directed mutagenesis, the efficiency for large-fragment DNA knockout was 23.6%, and the initial efficiency for simultaneous point mutation of three genes was approximately 50% (Liu et al., 2019). Wu et al. introduced a mutant NgAgo protein that enhances RecA-mediated homology-directed repair (HDR) efficiency into the *Francisella novicida* U112 CRISPR/Cpf1 system, constructing the CAMERS-B gene editing tool in *B. subtilis* 168, which achieved 100% efficiency for simultaneous mutation of six genes (Wu et al., 2020). The CCB-CIGE system established by Hao et al. achieved a double-gene knockout efficiency of 58.3%, future optimization of crRNA expression and HR mechanisms may further improve multiplex editing efficiency (Hao et al., 2020). The CRISPR-MAD7 system established by Laforge et al. enabled simultaneous modification of four genomic loci, combining gene knockout, gene insertion, and point mutation, while simultaneously removing overexpressed competence genes and gRNA coding sequences through HR (Laforge et al., 2025).

### 3.4 Base editing for multiplex editing

Multiplex gene editing technologies generally face the challenge of decreasing efficiency as the number of edited sites increases. CRISPR-Cas-based editing tools, due to the induction of DSBs or single-strand nicks, significantly reduce transformation efficiency and multi-gene editing efficiency. BEs, which do not rely on DSBs or HR, offer unique advantages in multiplex editing. Yu et al. established the first CRISPR-dCas9-AID base editing system in *B. subtilis* 168. After optimization, the single-plasmid system exhibited an editing window of 5 nucleotides, achieving 100% editing efficiency for three targets and 50% for four targets (Yu et al., 2020). To further improve multiplex editing efficiency, Kim et al. fused PmCDA1 and UGI to the C-terminus of dCas9 to construct CBE4. The optimal editing window of this system was positions 16–20 upstream of the PAM. After optimizing promoter expression levels, the simultaneous editing efficiencies for two and three targets both reached 100%, that for four targets reached 83.3%, and that for five targets reached 75.5% (Kim et al., 2021). Hao et al. integrated PmCDA1 and nCas9 into the genome of *B. subtilis* 168 and successfully broadened the editing window to eight nucleotides by extending the sgRNA sequence; the base conversion efficiency of the editor was effectively improved by adding an artificial stem-loop structure at the 3′ end of the sgRNA (Hao et al., 2021). Subsequently, they developed a dual BE by fusing the mutant TadA (ABE8e) and PmCDA1 to the N-terminus of nCas9, constructing the BS4 system. This system exhibited an editing window of 8–11 nucleotides, and in multiplex editing mode, the editing window and overall efficiency at each target site were generally comparable to those of single-site editing. Compared with single-deaminase BEs, the dual BE can efficiently generate more diverse *in vivo* mutant libraries, which is expected to facilitate the rapid cultivation of high-performance and stable bacterial chassis cells in biomanufacturing and biopharmaceutical fields (Hao et al., 2022). Later, leveraging the smaller steric hindrance of Cas12b compared to Cas9 and Cas12a, Hao et al. further engineered a CRISPR-Cas12b-based BE, termed dBhCas12b-based CBE (where dBhCas12b is a nuclease-deficient Cas12b from *Bacillus hisashii*). This novel BE exhibits a significantly expanded editing window, reaching approximately 19 nucleotides in *B. subtilis* and up to 43 nucleotides in *E. coli*, while maintaining low off-target effects and no apparent toxicity to the host strains (Hao et al., 2024). Building on this, they further developed a novel dual-function BE, MicroDFBEST, by fusing high-activity evoCDA1 and TadA9 to dBhCas12b. In *Escherichia coli*, this BE achieves an editing window of 26–33 nucleotides, representing the broadest range reported to date for microbial dual-function BEs. Although it has not yet been validated in *B. subtilis*, this tool holds great promise for use in this important industrial bacterium (Hao et al., 2026). To overcome the PAM dependence of BEs, Yan et al. used the nCas variant nSpRY to construct a set of efficient PAM-independent base editing tools in *B. subtilis*. After optimization, base conversion efficiencies reached up to 100% at highly active sites, while the editing window was precisely narrowed to 1–2 nucleotides. Furthermore, multiplex editing of two, three, and four genes was achieved (Yan et al., 2023).

### 3.5 CRISPR-associated transposases

Studies have shown that the CAST system can achieve efficient, RNA-guided large-fragment DNA integration and orthogonal editing in *E. coli* (Yang et al., 2021). Yang et al. applied the type I-F CAST system from *Vibrio cholerae* to *B. subtilis* 168 and confirmed that this system enables RNA-guided DNA transposition. However, the initially detected transposition efficiency was only 0.00018%. Proteomic analysis revealed that nearly all components were poorly expressed in *B. subtilis*. Even after optimizing culture conditions by lowering the temperature to 16 °C, the maximum efficiency only increased to 3.64%. Nevertheless, the CAST system remains a highly promising tool for large-fragment genome editing and awaits further optimization (Yang et al., 2023).

### 3.6 Summary

*B. subtilis*, as an important industrial chassis bacterial species, exhibits broad application prospects in industrial biomanufacturing by virtue of its GRAS status, efficient protein secretion capacity, and well-established fermentation processes. Reviewing the development history of its gene editing technologies, the field has progressed from the inefficient operations relying on long-fragment HR in the early days, through the transition to site-specific recombination systems such as Cre/*loxP*, to the paradigm shift brought about by the introduction of CRISPR-Cas9 technology in 2016. Currently, a multi-level, multi-functional toolkit centered on CRISPR-Cas9, CRISPR-Cpf1, nCas9, and BEs has been established. Nevertheless, several challenges remain: the efficiency of large-fragment integration (e.g., CAST system) needs improvement, some tools still rely on cumbersome plasmid elimination steps for iterative editing, and the PAM dependence and window control of BEs require further optimization. In the future, developing more efficient, simpler, and more universal editing tools targeting these issues will further unleash the potential of *B. subtilis* as a cell factory.

## 4 Gene editing in *C. glutamicum*

*C. glutamicum* is a core chassis bacterial species for the industrial production of bulk chemicals such as amino acids and organic acids. Reconstructing its metabolic network to enhance product synthesis capacity is an important goal in synthetic biology (Wang et al., 2023; Becker et al., 2018), and developing efficient and precise gene editing technologies is crucial for unlocking its industrial potential.

### 4.1 Traditional methods

Traditional gene editing methods in *C. glutamicum* are inefficient. Using suicide vectors carrying positive and negative selection markers (e.g., antibiotic resistance cassettes, *sacB* gene), gene cluster knockout and insertion can be achieved via double-crossover HR, but a single operation takes approximately 1 week (Schäfer et al., 1994). Suzuki et al. used the Cre/*loxP* system combined with I-SceI homing endonuclease-mediated DSBs to achieve scarless knockout of three independent large fragments (14 kb, 43 kb, and 10 kb) in *C. glutamicum* R, with editing efficiencies of 25–50%. However, this three-plasmid system required two rounds of transformation, and although large-fragment knockout was achieved, the process was even more time-consuming (Suzuki et al., 2005). Due to the low HR efficiency of *C. glutamicum*, improving recombineering efficiency became critical. Binder et al. screened five recombinases in *C. glutamicum* ATCC13032 and demonstrated that the RecET recombineering system exhibited the highest efficiency (Binder et al., 2013). Li et al. further evaluated seven recombinases, confirming that RecT showed the highest activity for ssDNA recombineering, while the RecET combination was most efficient for dsDNA recombineering. By optimizing the length and concentration of the repair template, editing efficiency was further improved (Li et al., 2021).

### 4.2 CRISPR-Cas systems: from Cpf1 to Cas9 and MAD7

In recent years, the introduction of CRISPR/Cas systems has greatly advanced the development of gene editing technologies in *C. glutamicum*. However, unlike other Gram-positive bacteria (e.g., *L. plantarum*, *L. lactis*, *B. subtilis*), the first CRISPR/Cas system applied in *C. glutamicum* was CRISPR-Cpf1. Studies have shown that active Cas9 is significantly toxic to *C. glutamicum* (Jiang et al., 2017; Cho et al., 2017). Jiang et al. were unable to obtain any transformants when SpCas9 was constitutively expressed in *C. glutamicum*. To address this issue, they constructed a CRISPR-Cpf1 system derived from *Francisella novicida* in *C. glutamicum* ATCC13032. This system exhibited no obvious host toxicity and enabled the introduction of small modifications such as point mutations with efficiencies of 86–100% (Jiang et al., 2017). In subsequent optimization, Zhang et al. determined that the optimal PAM sequence for *Fn*Cpf1 in *C. glutamicum* ATCC13032 was 5′-NYTV-3′, the optimal crRNA spacer length was 21 bp, and linear DNA could serve as a repair template (Zhang et al., 2019). Zhao et al. developed a multiplex gene editing method and a large-fragment chromosomal deletion strategy by optimizing the CRISPR/Cpf1-RecT system in *C. glutamicum* ATCC14067 (Zhao et al., 2020). Liu et al. combined endogenous recombinases with the CRISPR-Cpf1 system to establish a gene editing platform suitable for *C. glutamicum* S9114. Through sequence alignment and recombination efficiency validation, they screened the recombinase CauR from *Corynebacterium aurimucosum*, which exhibited the highest activity in *C. glutamicum* S9114. By optimizing Cpf1 expression, homology arm length, inducer concentration, and culture conditions, the gene knockout efficiency reached up to 77% (Liu et al., 2025). MAD7 is a Cas12a mutant derived from *Eubacterium rectale*, its smaller molecular weight offers advantages for large-fragment editing (Liu et al., 2020). Zhan et al. constructed a CRISPR/MAD7 system in *C. glutamicum* ATCC13032. After optimizing the promoter, replication origin, and PAM sequences, they achieved high-efficiency gene knockout with a broad range of recognizable PAM sites. The CRISPR/*Fn*Cpf1 system achieved 30% efficiency for knockout of a 20 kb fragment, whereas the MAD7 system reached 77.14% efficiency. Furthermore, the MAD7 system achieved 43.75% efficiency for knockout of a 25 kb DNA fragment (Zhan et al., 2024).

To overcome the cytotoxicity of SpCas9, researchers have adopted various codon optimization and expression regulation strategies. Zhang et al. performed *Streptomyces coelicolor* codon optimization of SpCas9 to reduce its expression level, thereby successfully achieving CRISPR-Cas9 gene editing (Zhang et al., 2020). Cho et al. performed actinomycetes codon optimization of SpCas9 and placed it under an inducible promoter (Cho et al., 2017). Liu et al. directly placed SpCas9 under an inducible promoter and constructed an effective gene editing tool (Liu et al., 2017). Peng et al. performed *C. glutamicum* codon optimization of SpCas9 and regulated its expression level using an inducible promoter, the constructed dual-plasmid system achieved up to 100% knockout efficiency for the *porB* gene, while also enabling efficient point mutation and gene insertion. This system has been extended for use in *C. glutamicum* CGMCC1.15647 (Peng et al., 2017).

In addition, several studies have combined CRISPR/Cas9 with recombineering for application in *C. glutamicum* (Cho et al., 2017; Liu et al., 2017; Wang et al., 2018; Yao et al., 2021). Liu et al. established an ssDNA recombineering-mediated CRISPR/Cas9 editing technology that enabled small-fragment modification and single-base point mutation of the *C. glutamicum* SL4 genome, with editing efficiencies exceeding 80.0%. Dual-site editing was also achieved in *C. glutamicum* ATCC13032 (Liu et al., 2017). Cho et al. developed a CRISPR/Cas9 coupled recombineering system by combining actinomycetes-codon-optimized Cas9 with RecT recombinase-mediated ssDNA recombineering. Using Cas9-sgRNA complexes for counter-selection of unedited strains, they successfully achieved scarless knockout of single genes and iterative multi-gene editing (Cho et al., 2017).

### 4.3 BE for multiplex and precision editing

DSBs introduced by CRISPR/Cas9 or Cpf1 significantly reduce bacterial cell viability, which hampers multiplex genome editing in bacteria (Zhao et al., 2020; Liu et al., 2017). BEs, which do not rely on DSBs, offer unique advantages in *C. glutamicum*. Wang et al. developed a multiplex automated *C. glutamicum* base editing method (MACBETH) using CRISPR/Cas9 and AID in *C. glutamicum* ATCC13032. This method required no exogenous DNA template and achieved efficient base editing in the absence of UGI, with efficiencies of up to 100% for single-site, 87.2% for dual-site, and 23.3% for triple-site gene editing (Wang et al., 2018). In a follow-up study, they used four Cas9 variants with expanded or altered PAM specificities to increase targeting range, and employed truncated or extended gRNAs to adjust the editing window (Wang et al., 2019). Deng et al. combined CBE with an adenine base editor (ABE) in *C. glutamicum* S9114 to construct a bidirectional base conversion tool, TadA-dCas9-AID, which achieved C → T, C → G, and A → G conversions within a 28 bp editing window (Deng et al., 2020). Heo et al. developed the CBE-STOP system for *C. glutamicum* ATCC13032, which achieved gene inactivation by introducing termination codons. The average single-gene editing efficiency reached 95.6% (at position C6) and up to 100%, and a succinic acid production strain BOL-1-STOP with five sequentially inactivated genes was successfully constructed (Heo et al., 2022). Zhang et al. combined SacB selection with a BE to simultaneously achieve dual-site mutation and fragment insertion (multiplex and multi-type editing) in *C. glutamicum* (Zhang et al., 2020).

### 4.4 Large fragment integration

As the application of CRISPR-Cas systems in *C. glutamicum* has expanded from single-gene editing to multiplex editing and large-fragment deletion, the challenge of low efficiency in large-fragment gene integration has become increasingly prominent (Jiang et al., 2017; Zhao et al., 2020; Liu et al., 2017; Wang et al., 2018). The bottleneck mainly lies in the low efficiency of HR (Wang et al., 2026). To address this challenge, researchers have established two main strategies: one is the use of CAST to bypass HR (Yang et al., 2023), and the other is the introduction of exogenous recombinases to enhance HR efficiency (Liu et al., 2025; Wang et al., 2026; Lobanova et al., 2022).

Yang et al. introduced the CAST system to *C. glutamicum* ATCC13032. By employing a spectinomycin resistance gene as a positive selection marker and the *sacB* gene for counter-selection to eliminate the plasmid, they achieved insertion of DNA fragments up to 9 kb at the target site with an efficiency of 13.4%, thereby enabling large-fragment integration (Yang et al., 2023). However, this method relied on selection markers and exhibited low efficiency, therefore, more active CAST systems need to be screened in the future to eliminate the dependence on selection markers.

Ye et al. established a Rec/ET-based iterative genome replacement system. First, a plasmid expressing RecET was transformed into *C. glutamicum* ATCC13032. Subsequently, nine large fragments (4–9 kb each) linked downstream to antibiotic resistance markers were iteratively transformed into the genome in successive rounds, achieving fragment replacement through HR. Using alternating selection with kanamycin and spectinomycin resistance genes, transformants were successfully screened, and ultimately replacement of a 55.1 kb fragment was achieved (Ye et al., 2022). Lobanova et al. developed the Extended Dual-In/Out strategy, which combined RecET recombination, phage ϕ16 site-specific recombination, Cre/*loxP* excision, Mu transposition, and chromosomal electroporation, successfully achieving integration of DNA fragments up to 10 kb in *C. glutamicum* ATCC13869 (Lobanova et al., 2022). Wang et al. combined RAGATH-associated DNA nucleases (RADs) with RecET, successfully inserting an 11.3 kb fragment with an editing efficiency of 13% (Wang et al., 2025).

For insertion of DNA fragments larger than 11.3 kb in *C. glutamicum*, the main bottlenecks are donor DNA preparation and delivery. Zhang et al. developed CACEXER, a large-fragment integration technology based on the *C. glutamicum* artificial chromosome (CAC), RecET recombination, and I-SceI digestion. The CAC carries the large donor fragment, I-SceI cleaves the CAC to release the linear large fragment, and RecET addresses the low natural recombination capacity of *C. glutamicum*. This method enabled single-step insertion of a ~ 50 kb synthetic fragment in *C. glutamicum*, and through nine iterative rounds, achieved precise replacement of a 361 kb genomic segment and construction of a semi-synthetic genome, with each round of insertion efficiency ranging from 22.2 to 100% (Zhang et al., 2025). Although this method allows large-fragment preparation and delivery using CAC, it involves a series of cumbersome steps: constructing the CAC vector via high-efficiency HR in yeast, transferring into *E. coli* for validation and plasmid preparation, and then transforming into *C. glutamicum*. A single round of editing takes 12 days. Wang et al. developed the One-Step Multi-Fragment Assembly Integration (OMAI) strategy, in which multiple PCR fragments with overlapping sequences are directly co-transformed into *C. glutamicum*, and a RecET mutant mediates *in vivo* assembly of the donor DNA and site-specific integration into the chromosome. A single round of operation takes only 3 days and achieves integration of 20.1 kb and 27.2 kb fragments, through iteration, 64.7 kb genomic replacement can be achieved. OMAI is expected to become the most efficient method for large-fragment insertion in *C. glutamicum* (Wang et al., 2026).

### 4.5 Summary

In summary, gene editing technologies in *C. glutamicum* have gradually evolved from early conventional methods relying on HR to editing strategies combining recombinases with CRISPR systems. They have further expanded to systems such as BEs and large-fragment genome integration, forming a relatively rich gene editing toolbox. Continuous efforts to address key issues, including Cas9 toxicity optimization, off-target correction of BEs, and efficient large-fragment integration, also provide feasible references for other Gram-positive bacteria that are difficult to manipulate genetically. Nevertheless, several challenges remain. The efficiency of CAST systems for large-fragment integration needs improvement. Although novel strategies such as OMAI are highly efficient, their general applicability requires further validation. BE efficiency under multiplex editing conditions still decreases as the number of targets increases. In the future, developing more efficient, simpler, and more universal tools to address these bottlenecks will further promote the development of metabolic engineering and synthetic biology in *C. glutamicum*.

## 5 Conclusions and outlook

**Figure 4:**
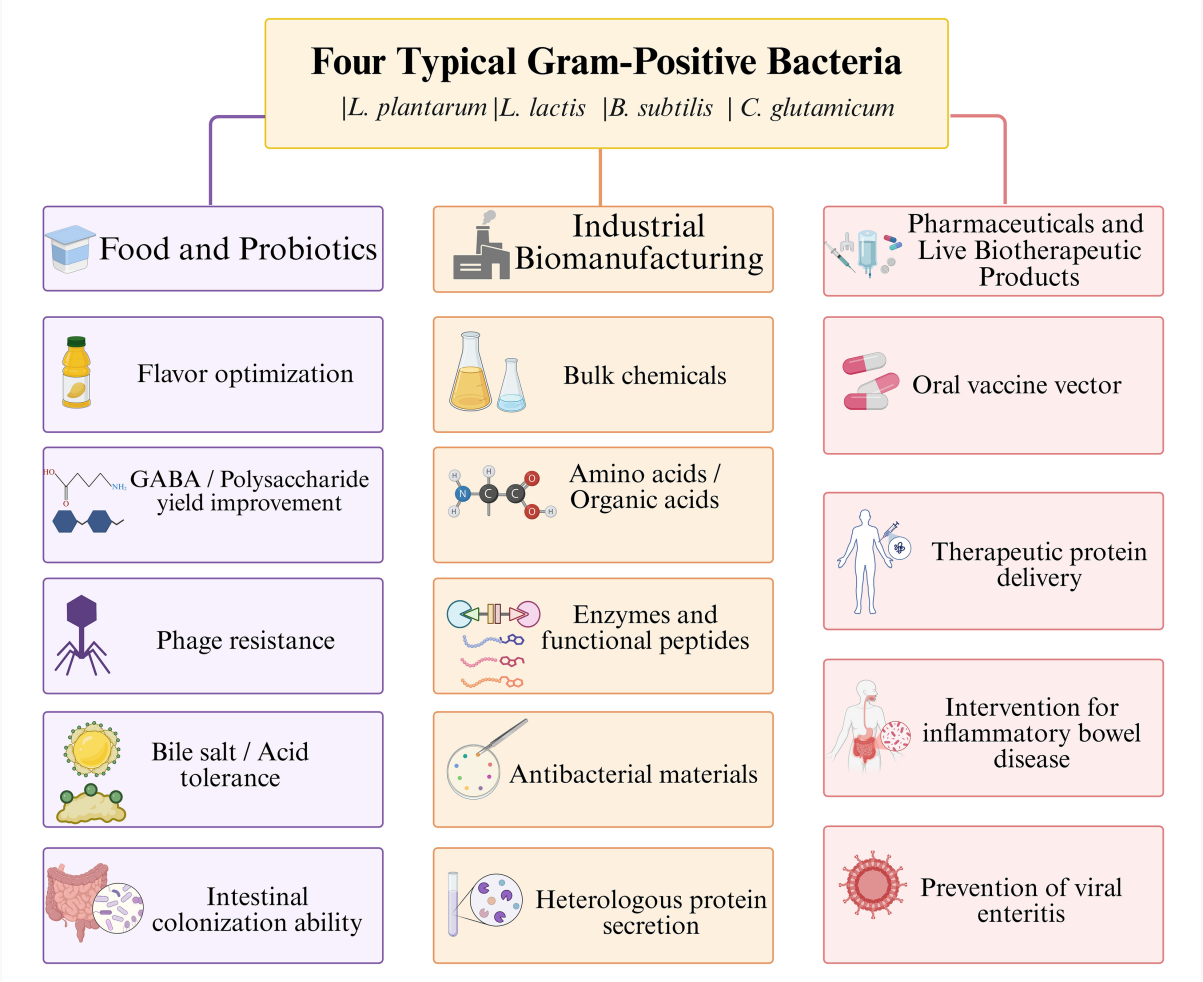
Representative applications of gene editing tools in four Gram-positive industrial bacteria. This schematic summarizes the main application scenarios of genetic engineering in four typical Gram-positive industrial bacteria (*L. plantarum*, *L. lactis*, *B. subtilis*, and *C. glutamicum*), divided into three major categories: food and probiotics; industrial biomanufacturing; and pharmaceuticals and live biotherapeutic products.

CRISPR-Cas gene editing technologies have been widely applied in four representative Gram-positive industrial bacterial species: *L. plantarum*, *L. lactis*, *B. subtilis*, and *C. glutamicum*. Table 1 summarizes the editing efficiencies obtained for different targets in different strains using various editing tools. The applications of these gene editing tools in the four representative Gram-positive bacteria are summarized in Figure 4.

**Table 1:** Gene editing tools and efficiencies of four typical gram-positive bacteria.

Species	Strain	System	Edit type	Target/fragment	Size	Efficiency	Donor DNA	References
*L. lactis*	*L. lactis* NZ9000	ssDNA Recombineering	Point mutation	*rpoB*	Four oligonucleotides	1%	ssDNA	van Pijkeren and Britton (2012)
	*L. lactis* NZ9000	CRISPR/Cas9	Knockout	*ldh*	945 bp	8–50%	Plasmid DNA	Song et al. (2021)
	*L. lactis* NZ9000	ssDNA recombineering + CRISPR/Cas9	Knockout	*noxD*	50/100 bp	100%	ssDNA	Guo et al. (2019)
			Insertion	*loxP*	34 bp	50%		
	*L. lactis* NZ9000	RecE/T-assisted CRISPR-Cas9	Insertion	*ldh*	4 bp	91%	Plasmid DNA	Zhou et al. (2023)
	*L. lactis* NZ9000	Single-BE	Point mutation	*LLNZ_02635 + LLNZ_08430*	–	100%	–	Tian et al. (2022)
	*L. lactis* IL1403	CRISPR-associated transposon system	Insertion	*lacZ*	1 kb	2 × 10^−4^	Plasmid DNA	Pechenov et al. (2022)
					10 kb	4 × 10^−5^		
*L. plantarum*	*L. plantarum* JDM1	dsDNA recombineering	Knockout	*gusA*	3 kb	75%	dsDNA	Yang et al. (2015)
	*L. plantarum* WCFS1	dsDNA/ssDNA recombineering + CRISPR/Cas9	Knockout	*nagB*	631 bp	53.3%	dsDNA	Zhou et al. (2019)
	*L. plantarum* WCFS1	RecE/T-assisted CRISPR-Cas9	Knockout	lp_0537, *lp_3662*, *lp_0082*	963 bp, 2,604 bp, 1,005 bp	50–100%	dsDNA	Huang et al. (2019)
	*L. plantarum* WCSF1	RecE/T-assisted CRISPR-Cas9	Insertion	*PspA-mCherry*	890 bp	50%	Plasmid DNA	Wiull et al. (2025)
	*L. plantarum* WCFS1	Single-BE	Point mutation	Dual-target, Triple-target	–	100%	–	Mitsunobu et al. (2025)
*B. subtilis*	*B. subtilis* 168	CRISPR/Cas9	Knockout	*amyE*	25.1 kb	89%	Plasmid DNA	Altenbuchner (2016)
				pulcherrimin biosynthetic gene	4.1 kb	97%		
	*B. subtilis* 1A751	CRISPR/Cas9	Knockout	*amyE*	–	100%	Plasmid DNA	Westbrook et al. (2016)
				*amyE **+** ugtP*	–	85%	–	
			Insertion	Hyaluronic acid biosynthetic operon	2.9 kb	69%	–	
	*B. subtilis* 168	CRISPR/Cas9	Knockout	*pps*	38 kb	80%	Plasmid DNA	So et al. (2017)
				*spo0A*	500 bp	97%		
			Point mutation	*sigE*	–	68%		
			Insertion	Green fluorescent protein gene	1.8 kb	97%		
	*B. subtilis SCK6*	CRISPR/Cas9	Knockout	*erm*	365 bp	100%	Plasmid DNA	Zou et al. (2022)
			Knockout	*amyE*	500 bp	100%		
			Point mutation	*spo0A*	–	100%		
	*B. subtilis* 168	CRISPR/Cpf1	Knockout	*pps* gene	38 kb	80%	Plasmid DNA	Hao et al. (2020)

	*B. subtilis* 168	CRISPR/Cpf1	Point mutation	*aprE + epr + nprE + bpr + mpr + nprB*	–	100%	Plasmid DNA	Wu et al. (2020)
	*B. subtilis* 168	CRISPR/MAD7	Point mutation	*amyE*	–	93%	dsDNA	Price et al. (2020)
				*gfpmut3*	–	100%		
	*B. subtilis* 168	CRISPR/nCas9	Knockout	*amyE*	1 ~ 8 kb	≥80%	Plasmid DNA	Liu et al. (2019)
			Insertion	*amyE*	1 ~ 2 kb	≥90%		
			Knockout	large DNA fragment	20.5 kb	23.6%		
			Point mutation	*amyE + upp + sigE*	–	49%		
	*B. subtilis* 168	Single-BE	Point mutation	*amyE(−18) + scoC(−18) + 5Cs*	–	100%	–	Yu et al. (2020)
				*mpr* + *nprB* + *vpr* + *wprA*	–	50%		
	*B. subtilis* 168	Single-BE	Point mutation	*sigF + srfAC, sigF + srfAC + aprE*	–	100%	–	Kim et al. (2021)
				*aprE + nprE + wprA + srfAC*	–	83.3%	–	
				*aprE + nprE + wprA + srfAC + sigF*	–	75.5%	–	
	*B. subtilis* 168	Single-BE	Point mutation	*secY + secE + secG*	–	Up to 100%	–	Hao et al. (2021)
	*B. subtilis* 168	Dual-BE	Point mutation	*bceB*	–	>60%	–	Hao et al. (2022)
	*B. subtilis* 168	PAM-free-BE	Point mutation	Dual genes	–	100%	–	Yan et al. (2023)
*B. subtilis* 168	CRISPR-associated transposon system	Insertion	*pyrE*	–	3.64%	Plasmid DNA	Yang et al. (2023)
*C. glutamicum*	*C. glutamicum* R	*Cre/loxP*	Knockout	Three separate large fragments	14 kb, 43 kb and 10 kb	25–50%	Plasmid DNA	Suzuki et al. (2005)
	*C. glutamicum* ATCC13032	ssDNA recombineering + CRISPR/Cpf1	Point mutation	*crtYf*	–	86–100%	ssDNA	Jiang et al. (2017)
	*C. glutamicum* ATCC 14067	CRISPR/Cpf1-RecT	Point mutation	*crtB+crtYf*	–	91.6%	ssDNA	Zhao et al. (2020)
	*C. glutamicum* S9114	*CauR*+CRISPR-Cpf1	Point mutation	*ldh*	–	77%	dsDNA	Liu et al. (2025)
	*C. glutamicum* ATCC 13032	CRISPR/MAD7	Knockout	CGP3 region	20 kb	77.14%	dsDNA	Zhan et al. (2024)
					25 kb	43.75%		
	*C. glutamicum* ATCC 13032	RecE/T-assisted CRISPR-Cas9	Point mutation	*gabT+gabP*	–	9.1–23.1%	ssDNA	Cho et al. (2017)
	*C. glutamicum* SL4	RecE/T-assisted CRISPR-Cas9	Knockout	*ldhA*	664 bp	47.3%	dsDNA	Liu et al. (2017)
	*C. glutamicum* ATCC 13032	CRISPR/Cas9	Knockout	*porB*	381 bp	100%	Plasmid DNA	Peng et al. (2017)

	*C. glutamicum* ATCC 13032	Single-BE	Point mutation	*upp, rfp*	–	100%	–	Wang et al. (2018)
				*upp + rfp*	–	87.2%		
				*upp + rfp + ald*	–	23.3%		
	*C. glutamicum* ATCC 13032	Single-BE	Point mutation	Single Gene (6 digits)	–	95.6%, up to 100%	–	Heo et al. (2022)
	*C. glutamicum* S9114	Dual-BE	Point mutation	*zwf*(C6)	–	30%	–	Deng et al. (2020)
	*C. glutamicum* ATCC13032	CRISPR-associated transposon system	Insertion	*crtYf*	9 kb	13.4%	Plasmid DNA	Yang et al. (2023)
*C. glutamicum* ATCC13032	RADs+ RecET	Insertion	*crtYe*	11.3 kb	13%	dsDNA	Wang et al. (2025)

All gene editing data are derived from the original literature, and editing efficiencies are influenced by multiple factors including host genetic background, target locus, edit type, and experimental conditions, therefore, direct cross-study comparisons are not appropriate.

Although these strains exhibit clear species-specific differences in genetic background, compatibility of editing components, and technological maturity, the technical challenges and optimization strategies encountered in the development of gene editing systems share common features. This section summarizes the findings from three perspectives: selection strategies for editing tools, cross-species common technical bottlenecks with corresponding solutions, and future directions in the field. The conclusions of this review are based on existing research data from the above four model strains. Although they cannot fully encompass the current status of gene editing system development for all Gram-positive bacteria, the design principles and optimization strategies summarized here may provide theoretical references for establishing gene editing systems in other difficult-to-engineer Gram-positive bacteria.

### 5.1 Strategies for selecting gene editing tools

The requirements for engineering industrial strains are diverse. They include small-fragment gene knockout or site-specific insertion, single-base precision mutation, large-fragment deletion or integration, and simultaneous multi-site editing. Rational selection of an editing strategy requires comprehensive consideration of host characteristics (transformation efficiency, Cas protein cytotoxicity, endogenous homologous recombination capacity), the length of the target fragment, and a balance between editing efficiency and experimental turnaround time. Based on literature data from the four representative bacterial species, this subsection describes the optimal tool selection for various editing scenarios (Figure 3).

#### 5.1.1 Knockout and insertion of small fragments

For single-gene or short-fragment knockout and insertion, CRISPR-Cas9/Cpf1 combined with recombineering is currently the most efficient and fastest approach. For example, in *B. subtilis*, a single-plasmid CRISPR-Cas9 system achieved knockout efficiency of 97% for a 4.1 kb fragment (Altenbuchner, 2016) and site-specific insertion efficiency of approximately 97% for the green fluorescent protein gene (So et al., 2017). In *L. lactis*, CRISPR-Cas9 combined with ssDNA recombineering enabled knockout of 50–100 bp fragments within 72 h with efficiency exceeding 75%, and when combined with RecET, the gene knockout efficiency reached 91% (Guo et al., 2019). In *L. plantarum*, CRISPR-Cas9 combined with RecET achieved single-gene knockout efficiencies of 50–100% (Huang et al., 2019). If the host is sensitive to Cas9 toxicity (e.g., *C. glutamicum*), CRISPR-Cpf1 or the MAD7 system is preferred. For example, MAD7 achieved a 77% efficiency for a 20 kb fragment deletion in *C. glutamicum* (Zhan et al., 2024). For DSB-free operations, BEs can also be used to introduce termination codons for gene inactivation. In strains where CRISPR-based gene editing systems have not yet been successfully established, traditional methods such as suicide plasmid-mediated double-crossover HR or Cre/*loxP* can be attempted.

#### 5.1.2 Point mutation and single-base editing

CRISPR-Cas gene editing systems can also achieve precise point mutations and single-base editing. However, BEs do not require DSBs or donor DNA templates and are therefore a better choice. For example, the Target-AID-based CBE in *L. plantarum* achieved single-target efficiency close to 100% and enabled triple-target editing (Mitsunobu et al., 2025). In *B. subtilis*, the CBE4 system achieved editing efficiencies of up to 100% for single, double, and triple targets (Kim et al., 2021). In *C. glutamicum*, the CBE-STOP system achieved up to 100% efficiency for single-gene editing (Heo et al., 2022). If the PAM restriction of a BE is too stringent, PAM-expanded variants (e.g., nSpRY) or CRISPR-Cas9-mediated ssDNA recombineering can be used. It should be noted that BEs have editing windows and off-target risks. The appropriate editor should be selected based on the distance of the target site from the PAM.

#### 5.1.3 Large-fragment knockout and integration

Large-fragment (typically >5 kb) gene editing remains a major bottleneck for Gram-positive bacteria. In *B. subtilis*, both the CRISPR-Cas9 system (So et al., 2017) and the CCB-CIGE system (Hao et al., 2020) can knock out a 38 kb gene cluster with 80% efficiency. In *C. glutamicum*, CRISPR-MAD7 achieved 43.75% efficiency for knockout of a 25 kb fragment (Zhan et al., 2024). For large-fragment integration, relying on HR is extremely inefficient. It requires the introduction of exogenous recombinases (e.g., RecET) or CAST. In *C. glutamicum*, the RecET-assisted OMAI strategy achieved integration of a 27.2 kb fragment within 3 days (Wang et al., 2026). This approach is highly efficient and time-saving, making it currently the best strategy for large-fragment insertion. Although the CAST system holds great promise, to date it has achieved only 9 kb insertion in *C. glutamicum* (13.4% efficiency), 3.64% efficiency in *B. subtilis*, and even lower efficiency in *L. lactis*. Thus, CAST systems still require further optimization. Overall, large-fragment knockout can be achieved in *B. subtilis*, but large-fragment insertion remains difficult. The large-fragment editing tools for *C. glutamicum* are relatively well developed and enable efficient editing. By contrast, large-fragment gene editing is more difficult in *L. plantarum* and *L. lactis*, and relevant tools still need further development.

#### 5.1.4 Multiplex editing

When editing multiple genomic loci simultaneously, the core challenges are the toxicity of multiple DSBs to the host and the resulting decrease in repair efficiency. The recommended solutions, in order of priority, are BEs (no DSBs, minimal host damage), nCas9 (generates only single-strand nicks), and CRISPR-Cpf1 (better compatibility). In *L. lactis*, CRISPR-cDBE enables multiplex editing (Tian et al., 2022). In *B. subtilis*, CBE4 achieves up to five-target editing (Kim et al., 2021). In *C. glutamicum*, the CBE-STOP system achieves simultaneous five-target editing (Heo et al., 2022), an nCas9-mediated editing system achieves simultaneous three-target editing (Liu et al., 2019), and the Cpf1-combined CAMERS-B system incorporating a mutant NgAgo protein achieves six-target simultaneous editing (Wu et al., 2020). It should be noted that multiplex editing efficiency generally decreases as the number of targets increases. It is advisable to design gRNA expression strategies rationally (e.g., using multiple crRNA arrays) and prioritize tools that cause minimal damage to the host.

### 5.2 Cross-species common bottlenecks and corresponding strategies

Although the four representative Gram-positive bacteria differ in technological maturity, the core bottlenecks exposed during their development are highly consistent. This section summarizes the existing bottlenecks from three perspectives: host endogenous repair capacity, cross-host incompatibility of editing tools, and inherent limitations of editing systems. Feasible improvement strategies are also discussed (Figure 2).

#### 5.2.1 Host endogenous DNA repair capacity

CRISPR-Cas gene editing systems rely on the Cas effector protein/sgRNA complex to create site-specific DSBs, thereby initiating recombination repair mechanisms. In eukaryotic cells, DSBs are typically repaired via the NHEJ pathway, whereas in most prokaryotes, repair depends on HDR. However, the genetic backgrounds of microorganisms are complex and variable. Different species exhibit different outcomes following chromosomal DSBs. For example, *Streptomyces* has high HDR efficiency and enables efficient HR-based CRISPR editing (Tong et al., 2015), whereas some *Lactobacilli* are highly sensitive to DSBs (Song et al., 2017). Even within the same species *L. plantarum*, endogenous repair capacities differ significantly (Leenay et al., 2019). The RecA-dependent HR efficiency of Gram-positive bacteria is generally low, which directly results in low efficiency of traditional gene knockout and CRISPR-mediated DSB repair. This problem is especially severe in multiplex editing, where the presence of multiple DSBs far exceeds the host’s repair capacity, causing multiplex editing efficiency to drop sharply as the number of targets increases (Zhao et al., 2020). Even when BEs are used to avoid DSBs, they only enable base conversion and cannot meet more complex editing needs. The use of nCas9 or Cpf1 reduces damage, but multiplex efficiency remains suboptimal.

Introducing recombinases (e.g., RecT, RecET, Redβ) enhances single-stranded and double-stranded DNA recombination capacity. Optimizing donor DNA design (e.g., increasing homology arm length, using single-stranded oligonucleotide donors) to match the host’s repair preferences can also improve recombination capacity and thus gene editing efficiency. Huang et al. combined the RecE/T recombinase system with the CRISPR system to successfully establish a CRISPR gene editing system (Huang et al., 2019). Wiull et al. constructed an inducible RecE/T-assisted CRISPR-Cas9 dual-plasmid system in *L. plantarum* WCFS1, further demonstrating that gene editing efficiency is positively correlated with recombinase expression level (Wiull et al., 2025). Therefore, combining recombinases with the CRISPR system to construct gene editing tools is an important strategy to overcome weak DNA repair capacity.

#### 5.2.2 Compatibility of gene editing tools across different species

The genetic backgrounds of microorganisms are complex and variable. Different gene editing tools exhibit different compatibility in different host cells. Cas protein toxicity is not a universal phenomenon, but it is prominent in *C. glutamicum* (Jiang et al., 2017) and some LAB (Huang et al., 2019), leading to failure in obtaining transformants. Furthermore, some systems that work efficiently in *E. coli* show a sharp decrease in efficiency or even fail completely in Gram-positive bacteria. For example, the CAST system achieves nearly 100% editing efficiency in *E. coli* (Gelsinger et al., 2023) and enables multiplex large-fragment editing (Zhang et al., 2020). In *L. lactis*, the editing efficiency for a 10 kb fragment is only 4 × 10^−5^ (Pechenov et al., 2022). In *B. subtilis*, even after optimization, the efficiency is only 3.64% (Yang et al., 2023). In *L. plantarum*, such systems have not yet been developed. This phenomenon is probably attributed to the poor expression of CAST components in Gram-positive bacteria. In addition, differences in host transformation efficiency and restriction-modification systems (Spath et al., 2012) are also important obstacles. Even for closely related strains, gene editing outcomes cannot be directly transferred from one to another (Leenay et al., 2019). Yang et al. identified and characterized a prophage P1 operon from *L. plantarum* WCFS1 that encodes three proteins mediating dsDNA recombination. However, they failed to find a similar operon in *L. plantarum* JDM1 (Yang et al., 2015).

To address the above compatibility issues, the following strategies can be adopted. Changing the nuclease type (e.g., using Cpf1 or MAD7) or performing codon optimization of Cas9 with low expression under an inducible promoter can alleviate Cas9 toxicity. Mining endogenous or cross-species CRISPR systems, CAST systems, and recombination systems is an important approach to solve tool compatibility. For example, in *E. coli*, Yang et al. discovered a CAST system from *Pseudoalteromonas translucida* KMM520, which achieved editing efficiency comparable to that of the reported *Vch*CAST system and enabled orthogonal transposition (Yang et al., 2021). Roberts et al. also characterized 10 previously uncharacterized type I-F3 CAST systems and Tn6677 (Roberts et al., 2022). More CAST systems can be identified through bioinformatics analysis to find candidates that better adapt to Gram-positive bacteria. Additionally, editing efficiency can be improved by optimizing culture conditions, promoters, and component expression levels. Overexpressing competence-related genes can improve transformation efficiency. Preparing plasmids from hosts with appropriate methylation patterns or treating DNA with *in vitro* methylases can help circumvent restriction systems (Wang et al., 2026).

#### 5.2.3 Technical limitations of existing gene editing tools

Although BEs do not require DSBs, their application is restricted by several inherent parameters. The editing window is typically only 5–8 nucleotides and is fixed in position (mostly located 10–20 bp upstream of the PAM). If the target base is not within this window, editing cannot be achieved (Mitsunobu et al., 2025). Most CBEs depend on UGI (Tian et al., 2022), which increases vector size and may affect efficiency in some hosts. Off-target effects are a common concern (Heo et al., 2022). The strict PAM sequence requirement further narrows the range of editable sites. Possible solutions include using bioinformatics tools to predict and exclude high-risk off-target sites, employing high-fidelity variants (with their activity verified in the target species), and performing whole-genome sequencing to validate off-target profiles when necessary.

### 5.3 Future trends

Although gene editing technologies for *L. plantarum*, *L. lactis*, *B. subtilis*, and *C. glutamicum* have made significant progress, these bacterial species still have shortcomings compared with well-established model bacterial species such as *E. coli*. These include a limited variety of editing tools, low efficiency of large-fragment integration and multiplex editing. Focusing on the existing pain points, future technological innovations can be advanced in three directions: mining of novel editing components, upgrading of large-fragment integration technologies, and artificial intelligence (AI)-enabled optimization of the entire editing workflow. These advances will further unleash the chassis engineering potential of Gram-positive industrial bacteria.

To exploit the application potential of CRISPR editing technologies in Gram-positive industrial bacteria, subsequent research needs to address the following core directions. First, continuous engineering of novel nucleases and BE tools remains fundamental to improving editing performance. This includes mining smaller Cas proteins (e.g., CasΦ, Cas12e), developing PAM-free or high-fidelity variants, and expanding the editing window and base conversion types of BEs (e.g., dual base editors, UGI-independent systems). Second, large-fragment integration remains a major bottleneck for gene editing in Gram-positive bacteria. Future efforts should focus on discovering novel CAST systems with better host compatibility through metagenomic mining and AI-assisted annotation, followed by modular engineering. Combining artificial chromosomes with CRISPR will enable multi-round iterative large-scale genome replacement while simplifying procedures and shortening turnaround times. Third, AI has been deeply applied to intelligent sgRNA design, off-target prediction, discovery of novel CRISPR systems, and engineering of editing proteins (Spath et al., 2012; Roberts et al., 2022; Kim et al., 2025; Li et al., 2025). Machine learning models such as CRISPRedit (Konstantakos et al., 2022), DeepFM-Crisp (Zhang et al., 2023), DeepCRISTL (Elkayam et al., 2024), CRISPR-GPT (Qu et al., 2026), and GLiDe (Xiang et al., 2025) will play key roles in predicting sgRNA efficiency, assessing off-target effects, discovering novel Cas proteins, and designing or optimizing existing editing tools. AlphaFold (Jumper et al., 2021) can accelerate the structural prediction and functional annotation of Cas proteins and methyltransferases. When combined with automated experimental platforms, these tools enable high-throughput automation of the entire design-build-analysis pipeline and continuously generate standardized data. In the future, integrating AI tools into experimental workflows will enable rational design of gene editing experiments, reduce trial-and-error cycles, and accelerate strain engineering.

Breakthroughs in the above directions will not only significantly enhance the gene editing capabilities of *L. plantarum*, *L. lactis*, *B. subtilis* and *C. glutamicum*, but also provide important references for tool development in other Gram-positive bacteria and even microorganisms that are difficult to manipulate genetically. Increasingly diversified, intelligent, and precise gene editing technologies will ultimately facilitate the creation of high-performance engineered strains and inject core driving forces into the fields of synthetic biology and biomanufacturing.

## Glossary

CRISPR: Clustered regularly interspaced short palindromic repeats
Cas9: CRISPR-associated protein 9
BEs: Base editors
CBE: Cytidine base editor
ABE: Adenine base editor
CRISPR-DBE: CRISPR-deaminase-assisted BE
CRISPR-cDBE: Cytidine deaminase-assisted base editor
CRISPR-aDBE: Adenosine deaminase-assisted base editor
HR: Homologous recombination
CAST: CRISPR-associated transposase systems
VchCAST: CAST system from *Vibrio cholerae*
LAB: Lactic acid bacteria
GRAS: Generally recognized as safe
DSBs: Double-strand breaks
SSBs: Single-strand breaks
ssDNA: Single-stranded DNA
dsDNA: Double-stranded DNA
nCas9: Cas9 nickase
dCas9: Catalytically dead Cas9
SpCas9: *Streptococcus pyogenes* Cas9
sgRNA: Single-guide RNA
AID: Activation-induced cytidine deaminase
PmCDA1: *Petromyzon marinus* cytidine deaminase
UGI: Uracil glycosylase inhibitor
INTEGRATE: Insert transposable elements by guide RNA-assisted targeting
CCB-CIGE: CRISPR/Cpf1 based-chromosome integration genome editing
HDR: Homology-directed repair
CAMERS-B: CRISPR/Cpf1 assisted multiple-genes editing and regulation system for *B. subtilis*
MACBETH: Multiplex automated *C. glutamicum* base editing method
RADs: RAGATH-associated DNA nucleases
CACEXER: CAC excision enhanced
CAC: *C. glutamicum* artificial chromosome
OMAI: One-step multi-fragment assembly integration
NHEJ: Non-homologous end joining
